# Diatoms: harnessing nature’s microscopic marvels for biosensing and multifaceted applications

**DOI:** 10.1007/s12551-024-01249-8

**Published:** 2024-11-05

**Authors:** Krupa Bhat, Maryam Abdul Ajees, Pawas Kumar, Vibha, Vyasraj G. Bhat, Roopa Nayak, Nirmal Mazumder

**Affiliations:** 1 Department of Biophysics, Manipal School of Life Sciences, Manipal Academy of Higher Education, Manipal, 576104 India; 2 Department of Radiation Biology and Toxicology, Manipal School of Life Sciences, Manipal Academy of Higher Education, Manipal, Karnataka 576104 India

**Keywords:** Diatoms, Biosensors, Drug delivery, Scanning electron microscope, Optical microscope, Marine biosensing, Photoluminescence

## Abstract

This article discusses the use of diatom in biosensing and various applications. A thorough understanding of the biosensing properties of diatoms is essential for the advancement of life sciences technologies. This review elucidates the emerging significance of diatoms in biosensing applications by highlighting the high surface area-to-volume ratio, biocompatibility, and facile functionalization of them. We examined the possible application of diatoms as flexible biosensing systems for the detection of various analytes, such as biological molecules, heavy metals, and poisons, by considering the findings of earlier research. Additionally, to show how diatoms can be used to create precise and sensitive biosensors, by integrating with several transduction modalities, including optical, electrochemical, and piezoelectric methods. We also delve into the potential applications of diatom-based biosensing in the future as well as pertinent topics such as repeatability, stability, and scalability. This provides an in-depth analysis of the quickly evolving field of diatom-based biosensing, which could affect several industries, such as environmental monitoring, food security and medical diagnostics.

## Introduction

Diatoms are unicellular microalgae of the class *Bacillariophyceae* found ubiquitously in marine and freshwater environments worldwide. These microscopic organisms are characterized by their unique cell walls, known as frustules, composed of silica (Squire et al. 2019; Mazumder et al. 2022). They are the only organisms on planet whose cell wall is composed of silica, giving them a transparent appearance under a light microscope (Mayzel et al. 2021). Diatoms are renowned for their intricate and ornate frustule structure, which can vary among species (Niu et al. 2023). Diatoms account for 20–30% of global oxygen production while also serving as food source for various organisms (Zgłobicka et al. 2021). The earliest fossilized evidence of diatoms can be found from the late Jurassic period (150–200 million years ago) of the extant genus *Hemiaulus* in Thailand (Squire et al. 2019). This review will delve into the intricacies of diatoms, exploring the importance of frustule structure in biosensing, wherein we will discuss medicinal benefits, environmental monitoring, and other applications. Research has proven that diatoms can produce electronic signals in response to alterations in their surroundings, thereby positioning them as auspicious candidates for biosensing devices (Zhang et al. 2012). Despite these encouraging findings, there is still a knowledge gap concerning the underlying mechanisms of diatom-based biosensing and its practical implementation. Diatoms, with half a million species, have a unique frustule structure made of SiO_2_, consisting of epitheca, hypotheca, valves, and girdle bands. Chemical modification using (3-aminopropyl)triethoxysilane (APTES) allows for diverse applications, such as biosensors and drug delivery. Their multilevel pore substructures enable drug chemisorption and photonic properties, enhancing signal applications. One key area of focus in recent research is the use of diatom frustules in surface plasmon resonance (SPR) biosensors (Rea and Stefano 2019) and fluorescence resonance energy transfer (FRET) (Marshall et al. 2012; Li et al. 2022). Diatom frustules, with their porous and nanostructured nature, offer a large surface area for biomolecule immobilization, enhancing the sensitivity of SPR and FRET biosensors. These biosensors can detect a wide range of analytes with high sensitivity and specificity. Researchers are exploring the use of diatom frustules in photonic crystal-based biosensors and microcavity resonators for label-free and extremely sensitive detection of biomolecules, offering unique interaction capabilities (Tisso et al. 2022). This approach enables the development of biosensors that can monitor cellular processes or detect specific biomolecules through fluorescence signals. Additionally, their fossilized remains, known as diatomaceous earth, have practical applications in industries such as filtration, agriculture, and even nanotechnology due to their unique structural properties. Understanding the historical development and significance of diatoms is crucial for understanding their ecological importance and technological applications in modern society (Mazumder et al. 2022).

### Morphology and structure of diatoms

**Fig. 1:**
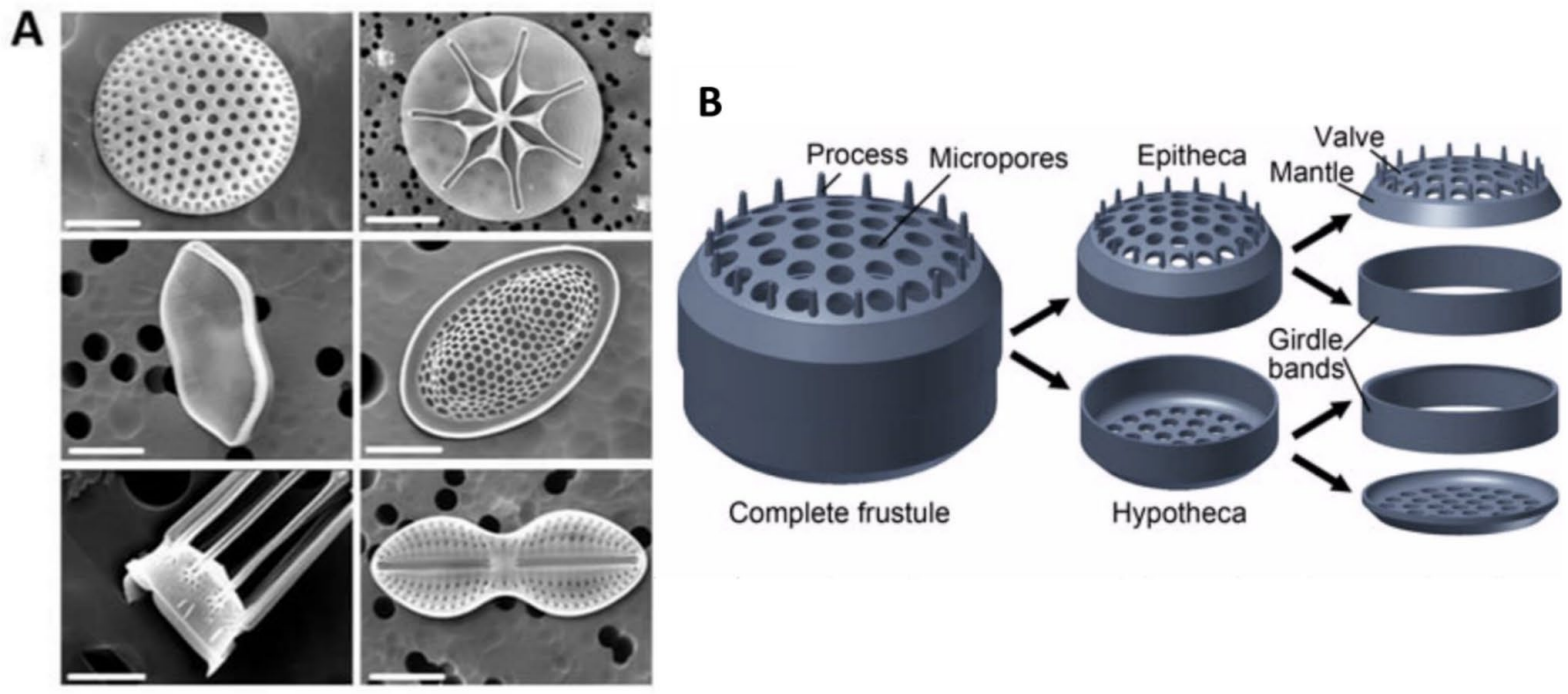
**A** SEM images of the cell walls of different diatom species. **B** Illustration of a centric diatom frustule with a cross-sectional profile of the silica wall typically formed by three overlapping porous layers: cribellum, cribrum, and areola. Reproduced with permission from Zhang et al. (2012); Terracciano et al. 2018

The morphology and structure are key characteristics that define diatoms, known for their intricate cell walls and diverse shapes. Diatoms are encased in a silica-based frustule, which consists of two overlapping parts: an epitheca and a hypotheca. These parts fit together like a petri dish, offering protection and support to the cell (Kröger and Wetherbee 2000). The frustule is punctuated with intricate patterns of pores and ridges, giving each species a unique appearance that aids in classification. The size, shape, and ornamentation of frustules are critical factors used to distinguish between different diatom species, making microscopy an essential tool for diatom identification and research. Understanding the morphology and structure of diatoms is fundamental to understanding their ecological role and evolutionary history, highlighting the significance of these microorganisms in marine ecosystems (Benoiston et al. 2017). In-depth studies of diatom morphology provide crucial insights into diatom adaptations and responses to environmental changes, underscoring the importance of continued research in this field. This phenomenon was studied by taking images from a scanning electron microscope (SEM) (as shown in Fig. 1) (Niu et al. 2023). The intricate hierarchical structure of diatom biosilica at the nanoscale has attracted attention as a template for material synthesis, especially regarding silicification under mild pH conditions and at room temperature (Patwardhan 2011). Early investigations in this field concentrated on employing polycationic peptides found in diatoms, known as silaffins, to chemically synthesize hybrid complexes of silaffin-silica from silicic acid (Kroger et al. 1999; Kröger and Poulsen 2008). This research has been expanded to create immobilized enzyme reactors by incorporating enzymes such as catalase, horseradish peroxidase, and butyrylcholinesterase into the reaction. Additionally, it involves attaching polyamines to lysines to replicate the interaction observed between silaffins and polyamines during silica precipitation from silicic acid (Luckarift et al. 2006; Naik et al. 2004; Wieneke et al. 2011). An enhanced understanding of the design principles governing the formation of biosilica cell walls in diatoms is expected to aid in the future in vivo bioassembly of genetically modified biosilica structures within transformed diatoms. This knowledge will also guide the bioinspired chemical assembly of silica-immobilized protein structures in vitro (Wang et al. 2009; Kent et al. 2009).

### Classification of diatoms

The classification of diatoms is primarily based on their morphology, with key characteristics including cell shape, size, and siliceous frustule structure. Diatoms are typically classified into two main groups based on the morphology of the frustule: centric and pennate diatoms. Centric diatoms are circular in shape, while pennate diatoms have long, rectangular or elliptical shapes. Within each group, further classification is based on the presence or absence of processes, the structure of the frustule, and the pattern of ornamentation (Yang et al. 2023). The frustules of diatoms possess intricate patterns and markings made of opaline silica, providing a unique and diverse visual repertoire for identification. Understanding the classification of diatoms is crucial for ecological studies because it enables the accurate identification and tracking of different species in various environmental samples, contributing to our knowledge of aquatic ecosystems and environmental health. The detailed examination of diatom structure and classification aids not only in taxonomic studies but also in biomonitoring efforts and paleoenvironmental reconstructions (Pedraza et al. 2017).

## Biosensors

**Fig. 2:**
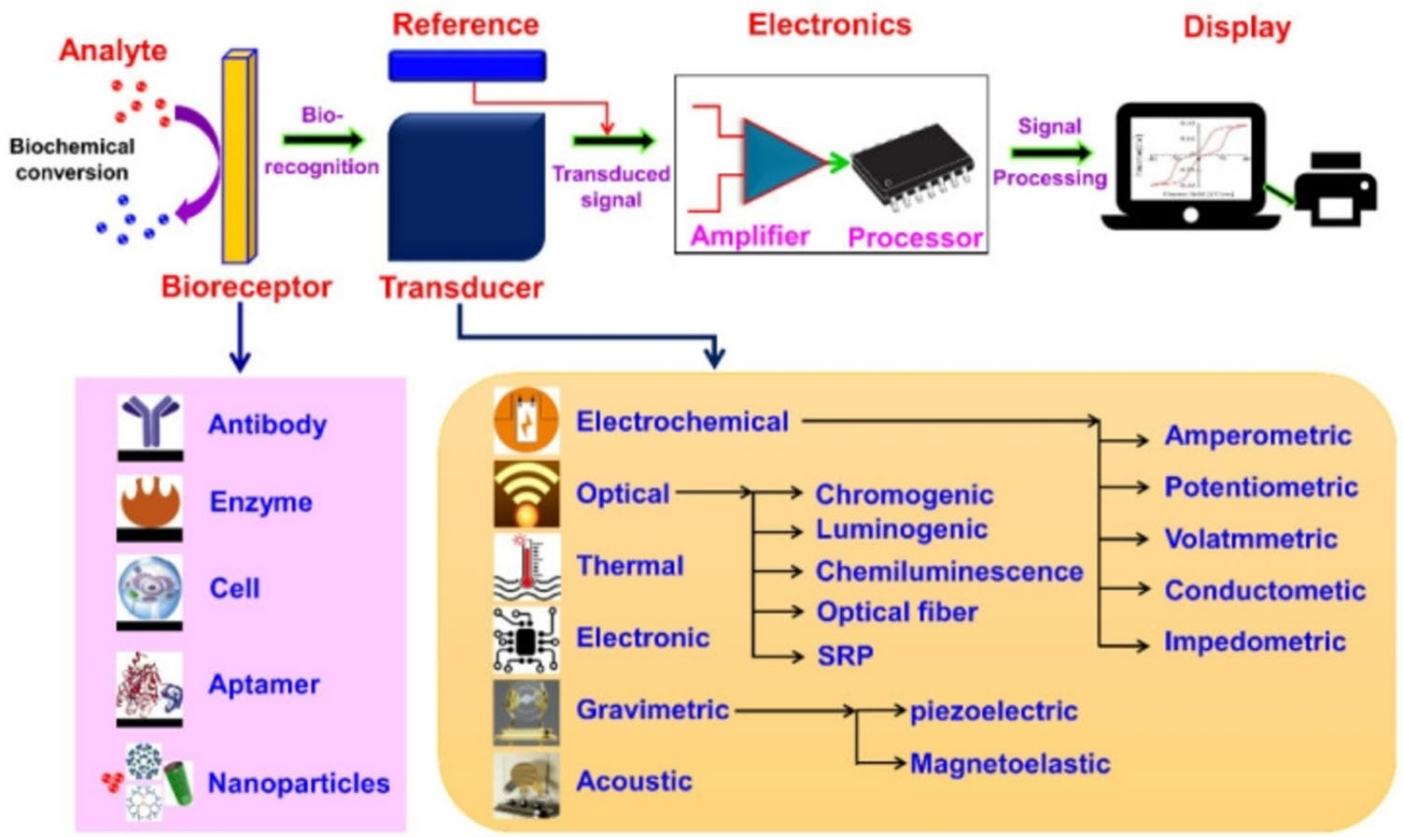
An example schematic representation of a bioreceptor, transducer, electronic system (processor and amplifier), display (PC or printer), and other forms of bioreceptors and transducers employed in the biosensors*.* Reproduced with permission from Naresh and Lee (2021)

A biosensor is a receptor-transducer device that combines a biological recognition element and a transducer to translate a biological response (e.g., antibody–antigen interaction) into an electrical signal (Kumari et al. 2023). The development of biosensors, materials, transducing devices, and immobilization procedures necessitates a multidisciplinary research strategy spanning various domains of knowledge, including chemistry, biology, and engineering. Biosensors are divided into two types based on their mode of operation: biocatalytic biosensors made up of enzymes and bioaffinity biosensors made up of nucleic acids, antibodies, and microorganisms (Mehrotra 2016). Biosensors can monitor and identify minute quantities of toxins, pH, and specific diseases. An analyte, a transducer, a bioreceptor, electronics, and a display are parts of a generic biosensor (this is schematically represented in Fig. 2).

### Components of a biosensor

A biosensor involves an analyte, a substance of interest, and a bioreceptor, a biomolecule, or biological element that recognizes the target substrate. The process of biorecognition generates signals, which can be in the form of microorganisms, light, heat, pH, charge or mass change, or tissue from plants or animals. A transducer is a crucial part of a biosensor, converting biorecognition events into electrical signals that can be quantified and linked to the quantity or presence of a chemical or biological target. The transducer amplifies and converts these signals into a digital format, which is then displayed using a user interpretation system, such as a printer or computer (Mehrotra 2016; Naresh and Lee 2021).

Biosensors have undergone significant miniaturization in the past few years. In keeping with these advancements, high-enzyme-producing microbial cells might be needed (Kazemi-Darsanaki et al. 2013). This is important, especially when replacing enzyme-based sensors with microbial cells. Microorganisms have been widely employed as biosensing elements in biosensor research due to their low cost, long half-life, and broad range of acceptable pH and temperature. Recent advancements in biosensor technology have propelled the development of highly sensitive and selective devices for diverse biomedical applications. For instance, nanotechnology has significantly enhanced biosensor performance, such as the bifunctional nanobiosensor designed for detecting chemokine ligands in colorectal cancer (CRC) cell lines (Chung et al. 2018). Additionally, nanomaterial-based strategies have improved enzymatic activities in biosensors, as seen in the peroxidase-like activity of dendritic nanochips for hydrogen peroxide determination in blood samples (Purohit et al. 2019). Furthermore, innovations in electrode materials, like nanotuned gold nanoparticles and graphene oxide, have enabled the rapid detection of sinapic acid and glucose in human saliva (Kumar et al. 2020), further enhancing the versatility and precision of nanotechnology-based biosensors for real-time diagnostics and health monitoring (Mokhtarzadeh et al. 2017). Additionally, advances in surface engineering, such as metallic dendrites and graphene oxide-chitosan nanocomposites, have also been pivotal in enhancing immunosensor performance for detecting biomolecules like carcinoembryonic antigens in clinical settings (Purohit et al. 2024). These developments underscore the ongoing evolution of biosensor technology towards more sensitive, rapid, and reliable analytical platforms for various practical applications.

## Diatom sensors and their applications

### FRET-based sensors

**Fig. 3:**
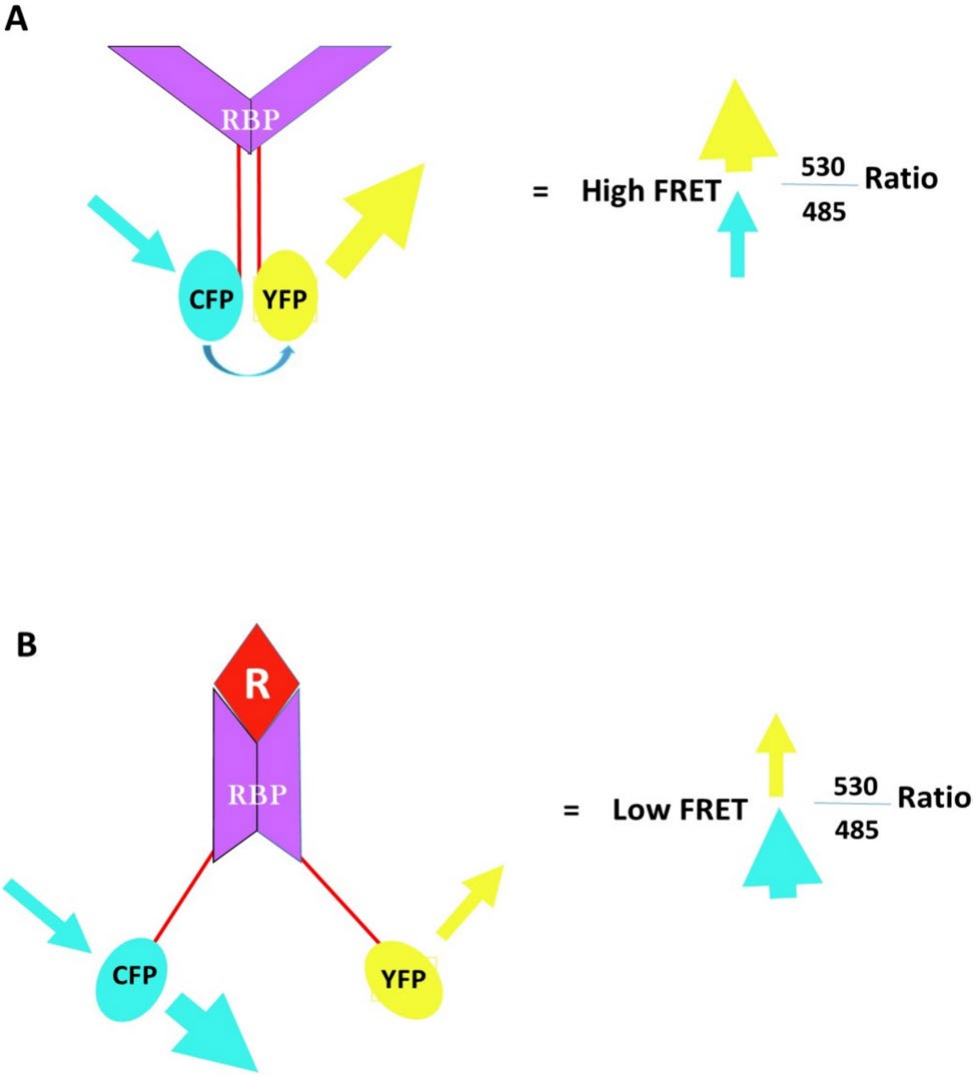
Schematic representation of ribose-induced conformational change in the recombinant chimeric protein, CRY to be used as FRET-based diatom biosensor. **A** In the absence of ribose, the RBP is in an open state. The proximity of CFP and YFP, which are connected to the amino and carboxyl termini of RBP, respectively, suggests this. When CRY is stimulated at 435 nm, *Y* is excited by the emission from C, increasing the 530/485 emission intensity ratio. **B** Ribose binding by RBP leads to a conformational change and increases the distance between CFP and YFP and decreases in energy transfer, thereby decreasing the yellow/cyan (530/485) emission intensity ratio. Consequently, when ribose concentrations increase, FRET decreases (Marshall et al. 2012)

Marshall et al. (2012) aimed to functionalize diatom biosilica using a FRET-based image biosensor for signaling without the need for reagents (Marshall et al. 2012). The fusion protein’s design incorporated a bacterial periplasmic ribose binding protein (RBP). The fusion protein was flanked by two fluorescent proteins, CyPet (CFP) and YPet (YFP), which operated as a FRET pair (Li et al. 2022). The structure and function of the recombinant fusion protein were first verified in proteins generated by *E. coli*. A 95% identity was found between the deduced amino acid sequence and the mass spectrometry data of CyPet-RBP-YPet (CRY). CRY showed typical ribose-dependent differences in FRET, with identical dissociation constants with and without an N-terminal Sil3 tag for biosilica targeting. The nanoarchitecture of a genetically modified biosilica cell wall allowed the complex Sil3-CRY fusion protein to function, demonstrating the potential of future materials with three-dimensional, hybrid, bio-assembled nanosystems with incorporated functional proteins (the mechanism of the protein is shown schematically in Fig. 3).

**Fig. 4:**
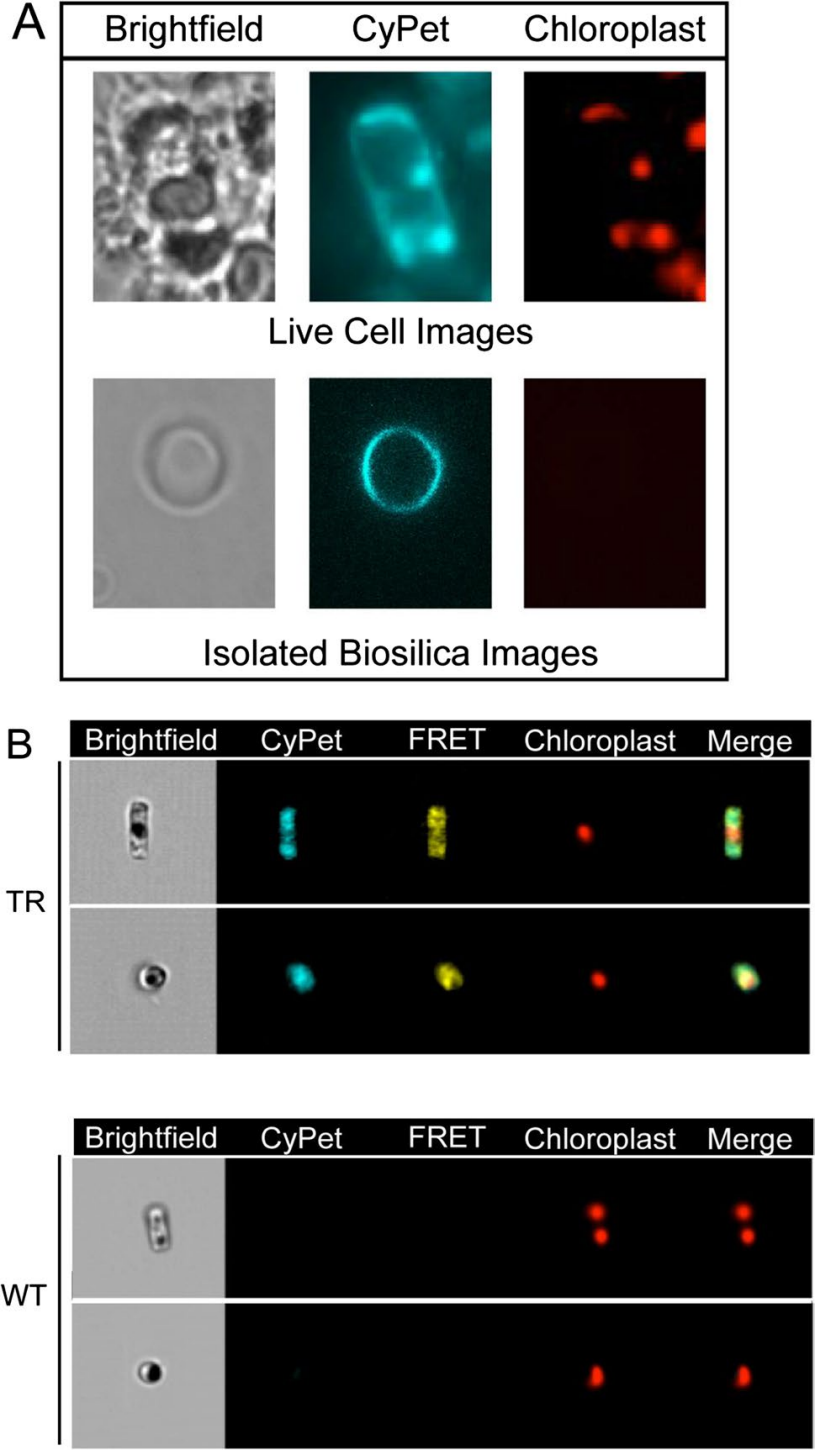
**A** Fluorescence microscopy: red autofluorescence and blue CyPet fluorescence were used to visualize the chloroplasts and biosilica, respectively, and brightfield microscopy was used to visualize the cell structure. Living transformed cells and isolated biosilica frustules were imaged. The absence of red autofluorescence in the isolated biosilica suggested the absence of chloroplasts, which suggested the absence of cellular material within the biosilica cell wall. Reproduced with permission from Marshall et al. (2012). **B** Imaging flow cytometry: pTpfcp/Sil3-CRY: fcp/nat-transformed (TR) and untransformed (WT) cells were captured on a camera using brightfield microscopy, and the yellow fluorescence of YPet from FRET, the cyan fluorescence of CyPet, and chloroplast autofluorescence were observed. To highlight the YPet and CyPet fluorescence that bordered the chloroplasts, images of cyan, yellow, and red were merged. Only red autofluorescence of the chloroplasts was observed in the WT cells. Reproduced with permission from Marshall et al. (2012)

The Sil3-CRY cassette was successfully transformed into a diatom expression vector, pTpfcp/Sil3-CRY: fcp/nat, which contained the nat gene and the Sil3-CRY expression cassette (Poulsen et al. 2006). Fluorescence microscopy and imaging flow cytometry confirmed the presence of recombinant chlorophyll in transformed diatoms and their isolated biosilica cell walls (Fig. 4A, B, respectively). The fluorescence was consistent with Sil3-CRY localization in the biosilica, which overlapped with the diatom’s perimeter. The bleed-through of red autofluorescence of chlorophyll into the cyan and yellow channels was responsible for the apparent colocalization of CRY in the chloroplast (Marshall et al. 2012).

**Fig. 5:**
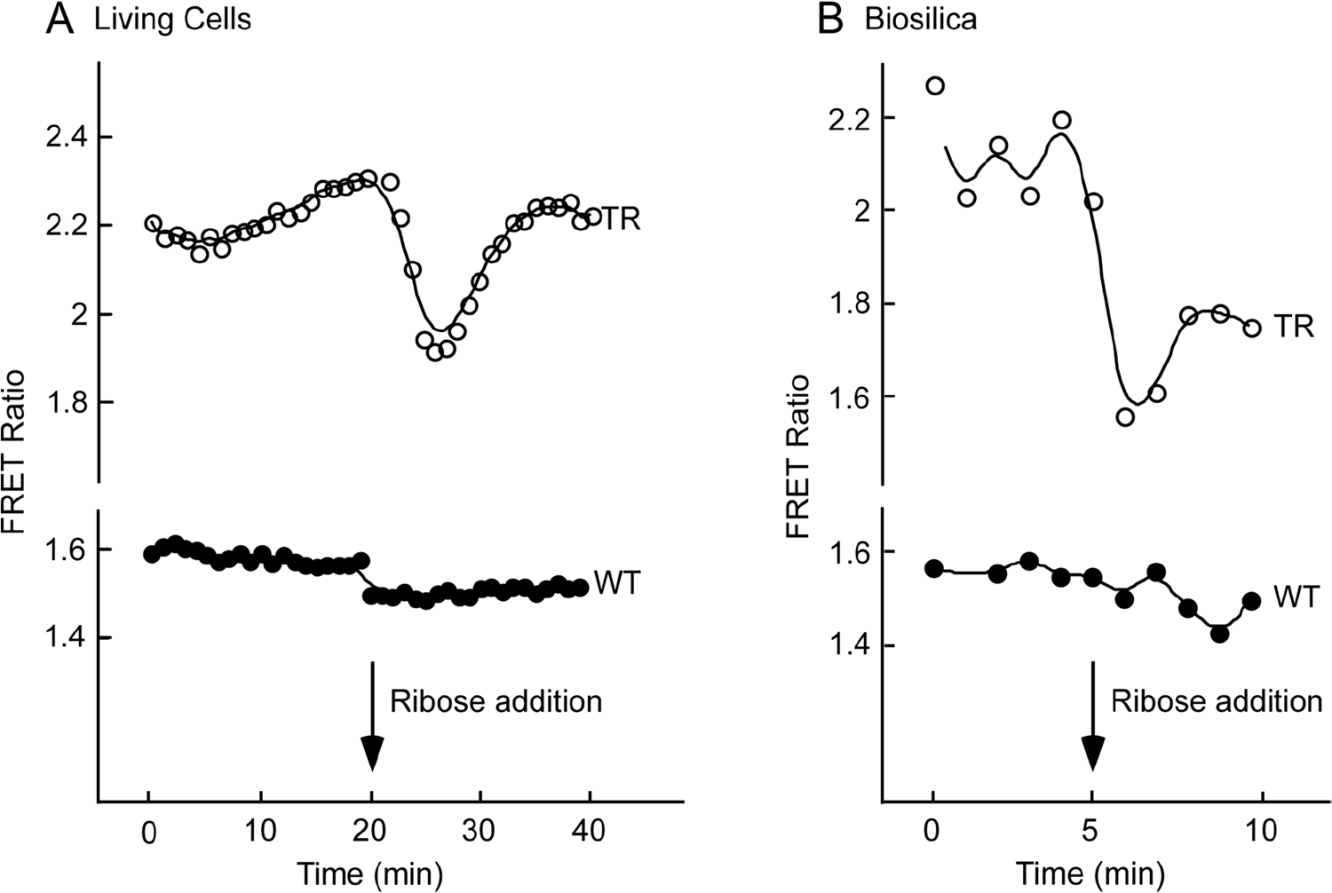
**A** The fluorescence of CyPet and YPet was imaged via time-lapse imaging of living cells both before and after the addition of 300 mM ribose. For 40 min, fluorescence images were taken concurrently in both fluorescent channels once every minute. After the first imaging, the cells were treated with ribose for 20 min. When ribose was added, the FRET ratio (530/485 nm RFU ratio) decreased (Marshall et al. 2012). **B** In a similar manner, biosilica cell walls were imaged throughout a 10-min period, and 300 mM ribose was added after 5 min. The addition of ribose causes a reduction in the FRET ratio. Reproduced with permission from Marshall et al. (2012)

Ribose-induced changes in FRET were observed in both living transformed cells and isolated biosilica cell walls. Initially, it was unclear whether the conformational changes in RBP required to drive changes in FRET would be constrained by the availability of substrate or steric hindrance caused by the immobilization of Sil3-CRY in the nanoporous architecture of the biosilica cell wall. (responses of representative cells are shown in Fig. 5A, B). At a saturating dose of 300 mM ribose, the FRET ratio (530/485 nm relative fluorescence units (RFU)) decreased by an average of 0.19 ± 0.01 (1 S.E., *n* = 10) and 0.43 ± 0.04 (1 S.E., *n* = 4) in transformed living cells and isolated biosilica cell walls, respectively (Marshall et al. 2012).

### Optical and optofluidic sensors

**Fig. 6:**
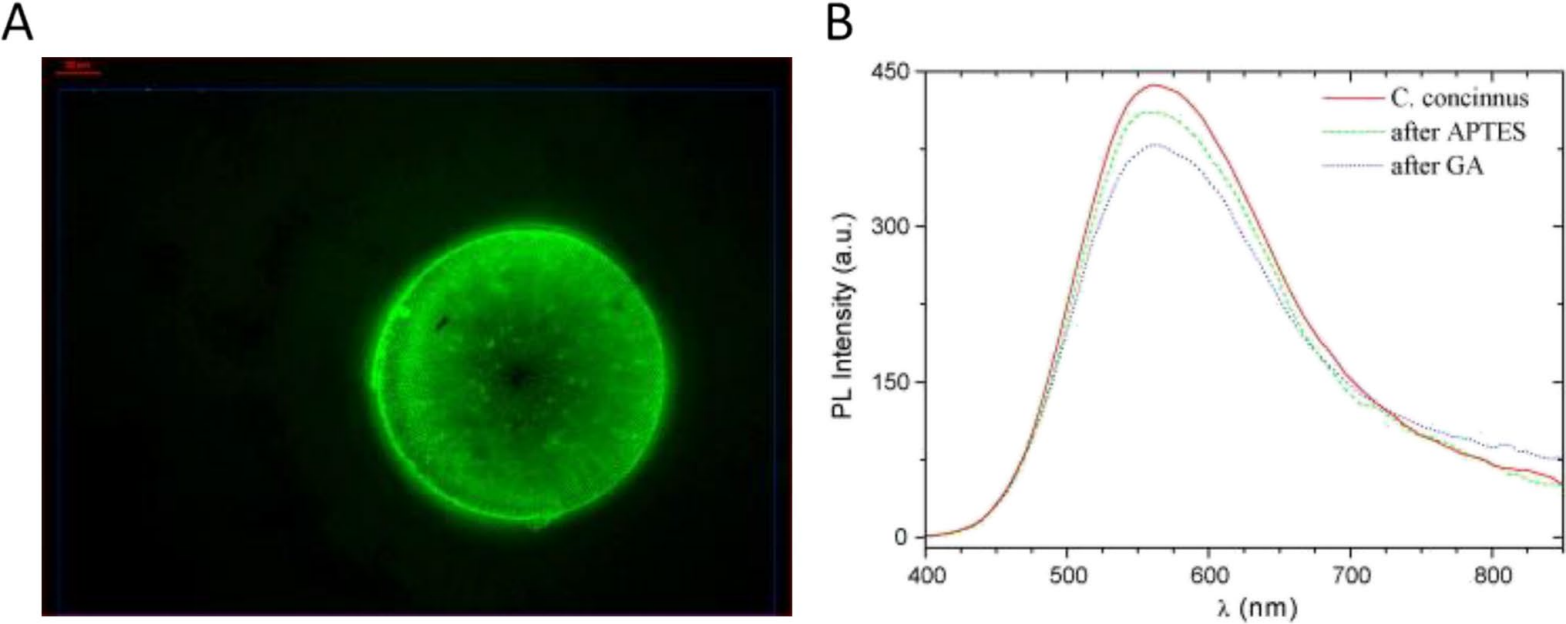
**A** Representative fluorescence image of a single diatom frustule under excitation by a high-power mercury lamp. **B** PL spectra after chemical diatom functionalization. Reproduced with permission from Krishna et al. (2021)

Marine silica, particularly from diatoms, has potential applications in optics, biophotonics, biosensing, filtration, and microfluidics. The green PL of diatom, peaking between 520 and 560 nm, is primarily attributed to Si–H groups. The macroscopic fluorescence image of a single diatom exposed to high-pressure mercury lamp radiation shows enhanced light emission through nanostructures. Additionally, the presence of nanostructures enhances light emission through quantum confinement effects (Fig. 6A) (Stefano et al. 2009). The PL spectrum of *C. concinnuc* diatom, shown in graph, peaks around 450–500 nm and demonstrates how chemical modifications affect PL intensity. The untreated diatom (red curve) exhibits the highest PL intensity, while surface treatments with APTES (green curve) and Glutaraldehyde (GA) (Blue curve) reduces the intensity. This decrease is consistent with the idea that chemical passivation alters surface recombination centers, reducing the light emission. The porous nature of diatom frustules and their ability to trap and stabilize fluorophores contribute to their strong initial PL, but modifications like these affect their optical properties, making them suitable for biosensor applications (Fig. 6B) (Stefano et al. 2009).

**Fig. 7:**
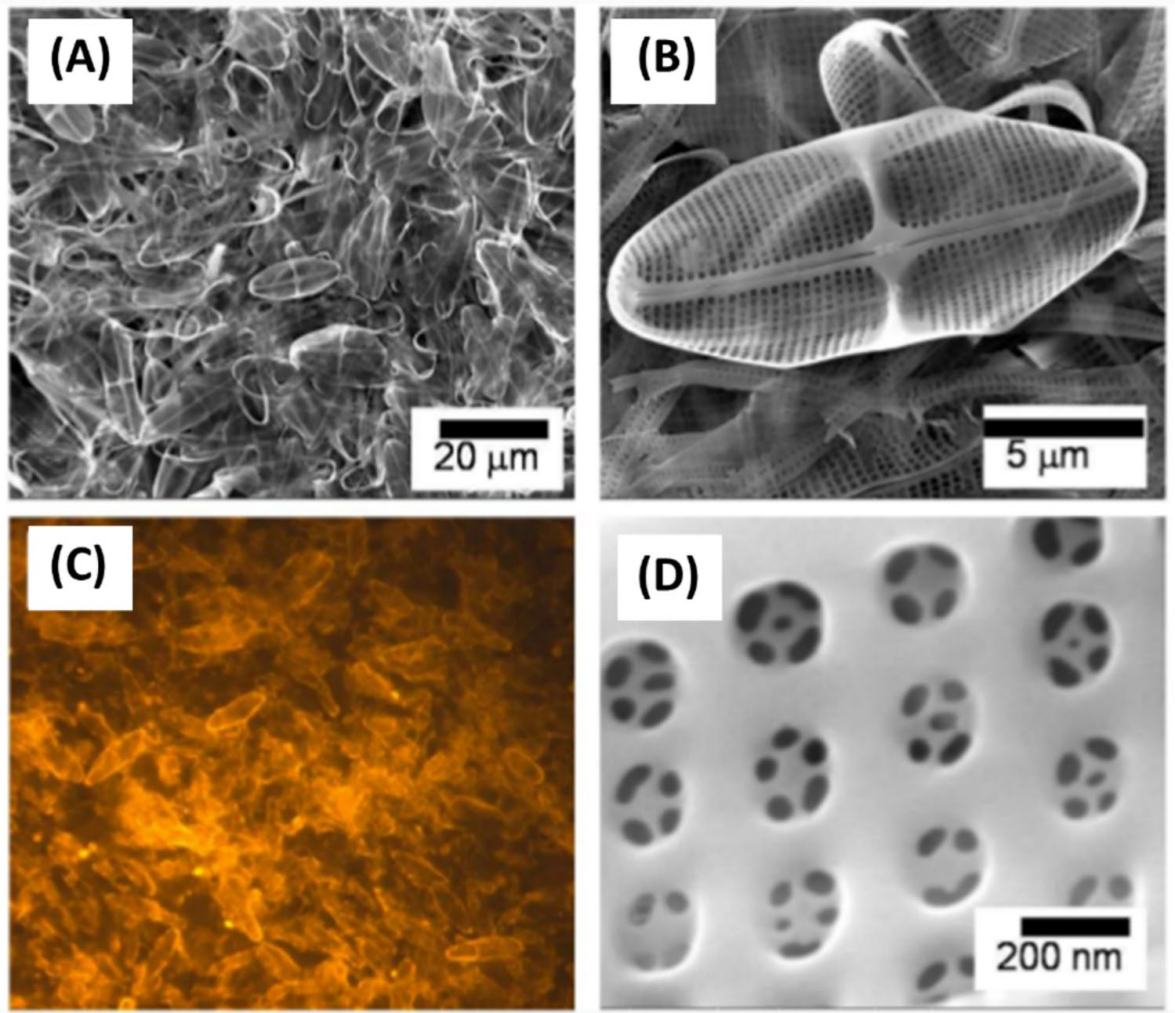
Imaging of *Pinnularia* frustules. **A**–**C** SEM images showing diatom frustule thin film layers, single-diatom frustules, and frustule pore arrays. **D** Epifluorescence Imaging of scFv-functionalized diatom biosilica after immunocomplex formation. Reproduced with permission from Zhen et al. (2016)

One of the works detailed the biological synthesis of Ge-doped biosilica frustules using a two-phase *Pinnularia* diatom cell culture, as well as its integration into an electroluminescent apparatus. The unique electroluminescent properties made possible by the Ge insertion and periodic pore structure of the frustules were highlighted in this study, which marks a first step towards the development of optoelectronic devices with cell-cultured components (Jeffryes et al. 2008a). FESEM images confirmed the oval-shaped frustules (~ 17 μm) of pennate diatom *Amphora* spp*.*, characterized by a ring-shaped girdle band and a porous structure with ~ 200-nm pores. Acid digestion effectively separated the valve and girdle band. Fourier Transform Infrared Spectroscopy (FTIR) spectra identified silica and surface hydroxyl groups, with subsequent amine functionalization altering PL, reducing blue emission due to surface passivation effects (Viji et al. 2014a). Fu et al*.* 2017 genetically expressed the enhanced Green Fluorescent Protein (eGFP) in *Phaeodactylum Tricornutum* diatoms, with eGFP acting as a chromophore capable of absorbing light in solar spectral regions where algal absorption is otherwise limited and transferring the collected light energy to the pigment-protein complexes of the photosystem units. This approach favored photochemical reactions, allowing to outperform the wild-type parental microalgal strain in biomass production rate under outdoor simulated sunlight conditions (Fu et al. 2017). PL-active biosilica frustules were isolated and modified from cultured cells of the marine diatom *Pinnularia spp.* using a single chain variable fragment (scFv) derived from an anti-troponin T (TNT) monoclonal antibody (Zhen et al. 2016). This approach facilitates the understanding of the optical characteristics of diatoms, such as the energy differential between the involved orbitals, which varies according to the fracturing structure of the diatoms. SEM images Fig. 7A–C of the *Pinnularia* diatom frustule reveal mostly complete structures with visible valves and girdle bands dispersed randomly on the surface. The frustule’s upper and lower theca separated as a result of the hydrogen peroxide treatment, exposing delicate biosilica features. *Pinnularia* is a pennate diatom with an elliptical shape with major and minor axes of around 20 μm and 6 μm. Its surface pattern consists of holes that are about 200 nm in diameter and are spaced 300–400 nm apart. Each larger nanopore contains four to five smaller ones that are about 50 nm in diameter.

Epifluorescence imaging serves as a crucial technique for investigating diatom biosilica functionalized with scFv following the formation of specific immunocomplexes. This method enables the detailed visualization of interactions between scFv and their target antigens, offering valuable insights into binding efficacy and the spatial localization of the functionalized biosilica within biological environments (as shown in Fig. 7D).

### Electrochemical sensors

A biosensor was designed using diatom silica as a template and integrated onto a microelectrode. Protein biomarkers were immobilized into the diatom pores using electrophoresis. The biosensor worked on the principle of electrochemical impedance spectroscopy and consisted of a silicon chip with gold electrodes covered by diatoms (Willner et al. 2003). The diatom silica membrane formed a high density of nanowells on top of each electrode, allowing for rapid and label-free detection of proteins in human serum samples (Lin et al. 2010; Vattipalli et al. 2011). The diatom *Coscinodiscus wailesii* with hierarchical pore structure was positioned onto the sensing sites, and immunoassay was performed for protein binding and detection. The detection range extended 6 orders of magnitude from 1 pg/mL to 1 μg/mL, much higher than enzyme-linked immunosorbent assay (ELISA) detection (Keshishian et al. 2009). The diatom silica membrane electrochemical biosensor showed enhanced sensitivity and selectivity, potentially used to detect disease biomarkers or proteins in clinically relevant concentrations. Diatoms can be used in electrochemical sensing by changing the parameters of gold electrodeposition used to fix antibody functionalized diatoms on metal electrodes, such as time, applied potential, and gold concentration.

### Biochemical sensors

Diatom frustules can be used as biochemical sensors, with fluorescent probes labeled on the silica shells. The silica deposition vehicle (SDV) facilitates this process, allowing for the immobilization of biomolecules on the surface of the diatoms for biosensing (Yang et al. 2011). Other biochemical sensors include Ge-doped diatom *Pinnularia* silica frustules, nanostructured TiO_2_, and antibody-functionalized diatoms. These sensors allow for efficient monitoring of biochemical and biomolecular interactions (Jeffryes et al. 2008b). Another biochemical sensor is antibody-functionalized diatoms, which detect immunocomplex formation. Marine diatom species like *Thalassiosira pseudonana* and *Phaeodactylum tricomutum* possess a far-red light sensing diatom phytochrome (DPH), which can use biliverdin as a chromophore and display red-shifted absorbance peaks. This far-red light sensing ability can help in biotic signal detection and molecular studies for solving the aquatic phytochrome conundrum (Fortunato et al. 2016). Diatom-based biochemical sensors have been used for the detection of nitroaromatic explosive derivatives through PL quenching due to the formation of the Meisenheimer complex (Selvaraj et al. 2018). Amine-functionalized frustules of diatom *Nitzschia* spp. were used for sensing 4-nitrotoluene (4-NT). These diatom biochemical sensors have a 1 μM limit of detection and are time and cost-effective. Enzyme tyrosine-immobilized diatoms modified with APTES can also be used for the detection and enzymatic removal of phenolic compounds in aqueous solutions (Pesavento et al. 2013). Diatoms can also be used for green biosynthesis of nanoparticles, which can be used as biochemical sensors for various applications.

### Microfluids-based sensors

Microfluidics-based diatom sensors involve the culture of diatoms like *Coscinodiscus excentricus* on a microfluidic chip made with Polydimethylsiloxane (PDMS) (Cai et al. 2013). The frustules are removed from the cells and bonded onto the PDMS microchamber using ultraviolet radiation. This method is advantageous as it makes the chemical reaction gentle without centrifugation or filtration, allowing the frustules to remain undamaged for functionalization (Lee et al. 2012). Soft lithography was used for fabrication, and the diatom solution was injected using micropumps. The diatom solution was then cultured in an incubator, and hydrogen peroxide was injected for washing and removal of organic matter (Cai et al. 2013; Zhang et al. 2012). An ultraviolet bonding technique was used to bond the clean frustules to the PDMS chamber, which did not destroy the nanoscale pore structures of the frustule. The fluorescence detected showed a high bonding strength in the sensor, enhancing the capture rate of detection and sensitivity. These diatom biosensors have hollow cylinders that store specific nanoparticles, which can be continuously released by the nanopores acting as filters for biosensing. They could also act as functional components in building complex 3D porous microchannels, improving the performance of microfluidics in molecular screening and biosensing (Cai et al. 2013).

### Immunoassay sensors

Immunoassay sensors based on diatoms, such as *Coscinodiscus concinnus,* are highly sensitive and cost-effective for point-of-care biosensing. These sensors use frustule modification by immunoglobin attachment to produce fluorescence upon antibody hybridization, allowing for the detection of various proteins. The fluorescence detection signal is 2.5 times higher than traditional protein sensors due to their higher surface area and high antibody density with multiple binding sites (Pan et al. 2012; Squire et al. 2018a). The spin-on-glass bonding technique was used to bond the SiO_2_ substrate and *C. concinnus* diatom together, resulting in high accuracy and high fluorescence detection (Stefano et al. 2008). The analyte molecule trapped on the sensor is then excited with a light source, resulting in a fluorescent optical signal captured by the camera. The amount of fluorescence detected is proportional to the analyte quantity, enabling analyte quantification. Photonic crystals can enhance fluorescence through enhanced emission and excitation due to fluorophores present on them. Diatoms, being natural photonic crystals, can be used in these immunoassay sensors, reducing fabrication techniques and improving fluorescence intensity and sensor sensitivity. There are various disadvantages of the traditional bonding techniques like anodic bonding, direct bonding, electrostatic adherence, and hydrofluoric acid (HF) bonding. Treatment with HF can destroy the diatom nanostructures which can lead to a decrease in the surface area and reduced sensitivity of the biosensor (Zhang et al. 2012). Immunosensors based on diatoms can also help in early detection of hormone N-terminal pro-B-type natriuretic peptide (NT-proBNP), which is secreted into the blood during heart failure and stress in the myocardial walls due to other cardiovascular diseases. This cost-effective method of early detection can help in heart failure diagnosis and enable early medical assistance. Immunosensors based on diatoms can also help in the early detection of Karnal bunt disease in wheat, as they are fast, economical, and specific. They can also be combined with Surface-enhanced Raman spectroscopy (SERS) detection based on diatoms *Pseudostaurosira trainorii* integrated with gold nanoparticles responsible for SPR to detect interleukin-8 (IL-8) in blood plasma. In conclusion, diatom-based immunoassay sensors offer ah cost-effective and efficient method for detecting various proteins and antibodies, making them valuable for point-of-care biosensing and medical diagnostics (Kaminska et al. 2017).

Recent advancements in diatom frustule modification, functionalization, and immobilization have led to successful applications in medicine, agriculture, and industry. These large surface areas, hierarchical porous nanostructures, make diatom frustules ideal for biosensor development, offering enhanced specificity and sensitivity, and allowing for flexible sensor design. Table 1 shows different diatoms utilized in bio-derived sensors and their applications. These diatom-based sensors are advantageous due to their low cost, rapidity of analysis, ease of use, eco-sustainability, and specificity (Leonardo et al. 2016). Diatom frustule modification for specific sensing properties requires strict control, involving silanization, chemical bonds, site-specific immobilization, and coating with metals and nanoparticles. Future research should merge diatom modification and biofunctionalization processes for significant advancements in using diatoms as bio-derived transducers and natural nanostructured supports in biosensing platforms.

**Table 1:** Different diatoms utilized in bio-derived sensors and their applications

Sensor	Diatoms	Wavelength	Technical details	Application	Reference
FRET-based Sensors	*Thalassiosira pseudonana*	*CFP-excitation-433 nm* *Emission-475 nm* *YFP-excitation-513 nm* *Emission-527 nm*	• CRY biosensor developed • Responds to ribose • Conformational changes detected • Generates FRET signals • Immobilization achieved	Ribose and other biomolecules can be detected without the need for any additional reagents	(Marshall et al. 2012; Li et al. 2022; Poulsen et al. 2006)
Optical and optofluidic sensors	*Amphora* spp.	*Excitation-370 nm* *Emission-440 nm*	• On-chip chromatography-SERS • Target compound identification • Human plasma analysis • Plasmonic modification application • Diatom biosilica use • Biomedical research potential • Diagnostics application potential	Detection of gases like NO_2_, single-molecule detection of trinitrotoluene, label-free detection of biomolecules like bovine serum albumin (BSA) proteins, early detection of *Salmonella typhi* aiding in environmental monitoring and clinical diagnostics of the water-borne illness typhoid, and detection of ultra-small analyte solution volumes in chemical analysis, environmental protection, and biomedical diagnosis	(Stefano et al. 2009; Jeffryes et al. 2008a; Viji et al. 2014a; Fu et al. 2017; Zhen et al. 2016; Kong et al. 2016; Viji et al. 2014b; Jarrett et al. 2020)
	*Thalassiosira rotula*	*Excitation-325 nm* *Emission-in air-533.4 nm* *In acetone-542.9 nm*			
	*Phaeodactylum tricornutum*	*Excitation-488 nm* *Emission-710 nm*			
Electrochemical Sensors	*Coscinodiscus wailesii* *Phaeodactylum tricornutum*	*-----------------*	• Nanoporous Si-ZrO_2_ composite • High selectivity demonstrated • Interference tolerance • Wide linear range • Concentration range: 3.4–64 μM • Suitable for real samples	Methyl parathion detection, disease indicators for determining cardiovascular risk, and label-free protein detection of human serum samples, surpassing traditional methods like ELISA	(Willner et al. 2003; Lin et al. 2010; Vattipalli et al. 2011; Keshishian et al. 2009; Gannavarapu et al. 2019; Liao et al. 2014; Dolatabadi and Guardia 2011; Panwar and Dutta 2019)
Biochemical Sensors	*Pinnularia* spp. *Coscinodiscus* spp. *Thalassiosira pseudonana* *Phaeodactylum tricomutum* *Nitzschia* spp. *Thalassiosira pseudonana* *Amphora* spp.	*Excitation and emission–650 nm*	• Diatom frustules functionalization • APES enhances detection • Improved PL • Low detection limit • BSA protein detection	The identification of nitroaromatic explosive derivatives such as TNT; the detection of dissolved ammonia; the use of dye-sensitized solar cells; the use of photocatalysts for the breakdown and sensing of toxic chemicals; the investigation of silica morphogenesis; soft lithography enhances biosensing in biomedical and environmental applications by providing precise fluid control, strong bonding, efficient molecular capture, and nanopore-mediated nanoparticle release	(Yang et al. 2011; Jeffryes et al. 2008b; Fortunato et al. 2016; Selvaraj et al. 2018; Pesavento et al. 2013; Chetia et al. 2017; Gale et al. 2009; Wang et al. 2021)
Microfluidics-based Sensors	*Coscinodiscus excentricus*	*Excitation and emission–380 nm*	• Diatoms optimize fluid flow • Microfluidic environment integration • Improved movement efficiency • Enhanced molecule manipulation • Microscale fluorescent molecules	Biomolecule detection; as functional components in the construction of complex 3D porous microchannels; in molecular screening; storing nanoparticles for release during sensing; and integrating *Coscinodiscus excentricus* frustules onto PDMS microchambers, which improve biomedical diagnostics, environmental monitoring, and molecular biology research by providing precise fluid control and biosensing capability	(Cai et al. 2013; Lee et al. 2012; Zhang et al. 2012; Cicco et al. 2015)
Immunoassay sensors	*Coscinodiscus concinnus* *Pinnularia* spp. *Cyclotella* spp. *Navicula lundii* *Pseudostaurosia trainorii*	*-----------------*	• *Cyclotella* spp. biosilica • Functionalized with immunoglobulin-G (IgG) • Increased PL • Sixfold enhancement • Antibodies attachment • Goat antirabbit IgG • Fourfold further increase	Analyte molecule detection and quantification, point-of-care fluorescence microscopy diagnostics, cellphone-based biosensors for point-of-care diagnosis in developing and underdeveloped nations, early identification of NT-proBNP in human plasma to aid in the diagnosis of heart failure, identification of IL-8 infection markers in blood plasma, and early identification of Karnal bunt disease in wheat	(Pan et al. 2012; Squire et al. 2018a; Stefano et al. 2008; Kaminska et al. 2017; Mishra et al. 2020; Squire et al. 2018b)

## Application of biosilica diatoms in medicine: a natural tool for targeted therapies (drug delivery)

### Biocompatible carriers of anticancer therapeutics

**Fig. 8:**
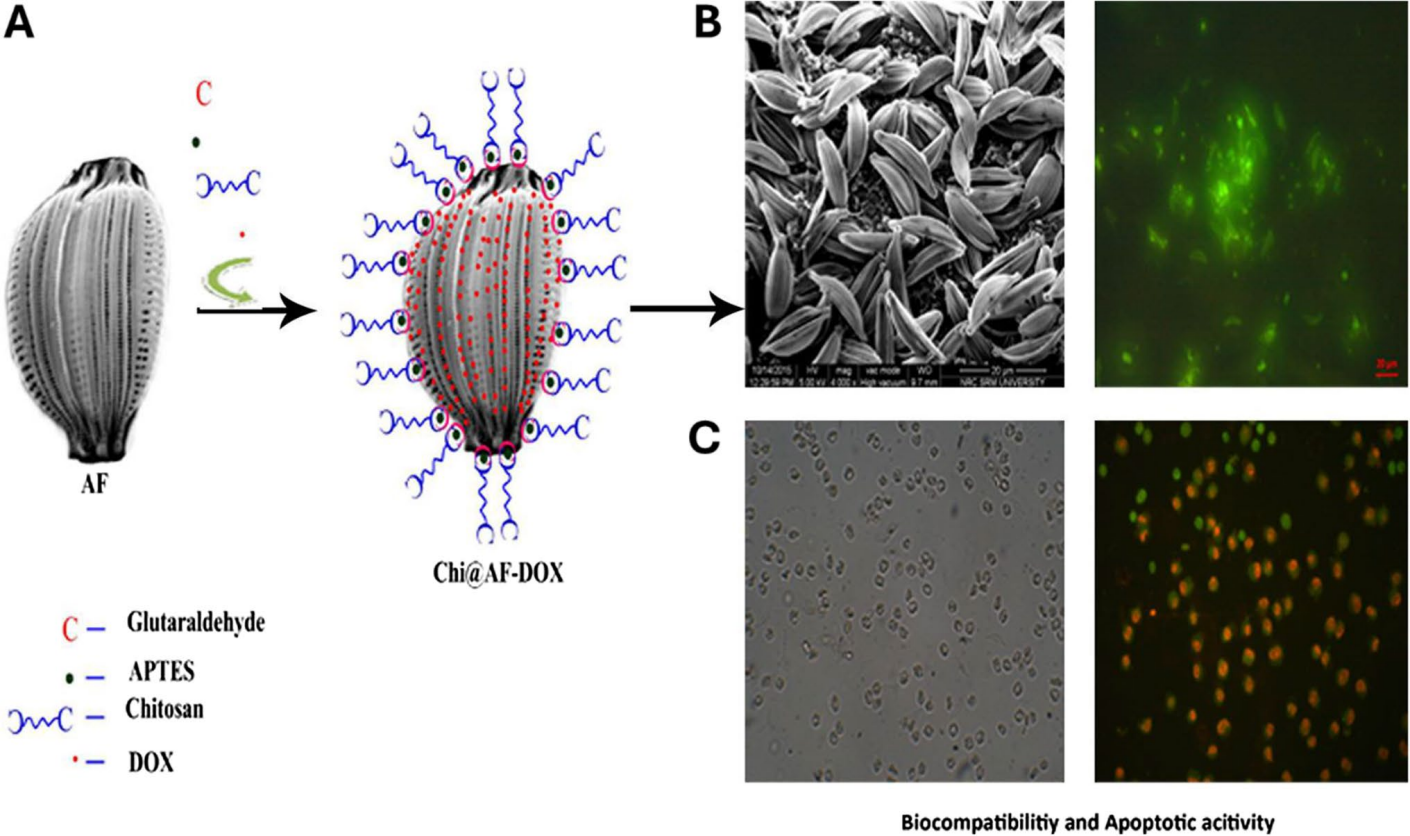
**A** Left schematic depicting the surface modification of *Amphora subtropica* frustules involving chitosan functionalization and subsequent Doxorubicin (DOX) loading (as described by Sasirekha et al.). **B** Microscopy image of fluorescein isothiocyanate-labeled diatoms captured using both FESEM and fluorescence microscopy (scale bar: 20 µm). **C** Combined bright-field and fluorescence microscopy image of A549 human lung cancer cells after exposure to chitosan-grafted/DOX-loaded Amphora subtropica. Orange fluorescence indicated cells undergoing apoptosis (programmed cell death). Reproduced with permission from Sasirekha et al. (2019)

Kröger and Voelcker’s work provided a notable illustration of the use of natural diatom biosilica to deliver anticancer drugs. They utilized genetically modified biosilica from the diatom species *Thalassioriaceas,* named *T. pseudonana,* to deliver hydrophobic anticancer medications such as camptothecin and 7-ethyl-10-hydroxy-camptothecin to neuroblastoma and Burkitt lymphoma HR1K cells. The genetic modification of *T.pseudonana* involves the integration of the IgG-binding domain of protein G (GB1-bearing fusion protein) into the diatom genome. This modification allowed the attachment of the GB1 fusion protein to the biosilica surface, facilitating IgG binding and subsequent integration of targeting antibodies. Following intraperitoneal injection of drug-loaded biosilica, tumor regression was observed in a neuroblastoma xenograft mouse model (Delalat et al. 2015). This study highlights a distinctive case of genetically modified diatoms in the realm of drug delivery, contrasting with other reports that concentrated on chemical surface modifications of diatoms to improve cellular targeting, adhesion, and drug loading/releasing efficiency (Delalat et al. 2015). For example, Terracciano et al. functionalized diatomite nanoparticles with polyethylene glycol and a cell-penetrating peptide and assessed their compatibility with blood, toxicity, uptake by cancer cells, and potential to administer the poorly water-soluble anticancer medication sorafenib to breast cancer cells (MCF-7 and MDA-MB-231). Researchers have discovered that modified nanoparticles exhibit low toxicity, optimal cellular uptake, and effective drug loading and release (Terracciano et al. 2015) (as shown in Fig. 8).

Using chitosan, Sasirekha et al. transformed the surface of the diatom *Amphora subtropica*. They demonstrated how the porous sections of the modified diatoms contained an encapsulation of the chemotherapeutic medication DOX. When utilized as a drug delivery system for anticancer treatment, this construct showed bright luminescence, prolonged drug delivery, biodegradability, biocompatibility, high drug loading, and lower toxicity than free DOX (Sasirekha et al. 2019). Kabir and his partners utilized a similar silanization-based chemical method to modify the surface of diatomaceous earth microparticles. Researchers have created core–shell materials that may be utilized to divide the compartments of a single drug delivery vehicle and encapsulate two different chemical medications at consistent molar ratios: DOX, which is hydrophilic, and paclitaxel, which is hydrophobic. The medication was loaded into the diatom core versus the outer cyclodextrin shell (Kabir et al. 2020). While chemical modification of diatom surfaces is a common method for drug delivery, another alternative involves the use of diatom frustules as templates or scaffolds to create biocompatible materials with ordered structures. Maher et al. described the synthesis of luminescent degradable silicon replicas of diatoms for the delivery of Daunorubicin (DNR) and DOX. They prepared silicon nanoparticles (SiNPs) through a modified magnesiothermic reduction process in which Mg reacts with silica to form a composite of MgO and Si. The acid treatment removed the MgO layer, leaving behind a silicone replica with a high surface area. The silicon diatom replicas demonstrated high crystallinity and improved degradation rates compared to those of the original diatoms, allowing for the prolonged and sustained release of DNR and DOX for up to 30 days. Confocal microscopy revealed that the replicas emitted red luminescence at 682 nm when excited at 458 nm (Maher et al. 2015, 2016). This study explored the potential of diatomite replicas as self-reported drug delivery systems. These replicas possess unique luminescence properties that are masked upon drug loading and reappear upon release, providing a real-time mechanism for monitoring drug delivery efficacy (Entwistle et al. 2018). *In vitro* studies confirmed sustained and pH-dependent drug release from these carriers, demonstrating greater cytotoxicity against cancerous cells in comparison with the medication in its free form.

**Fig. 9:**
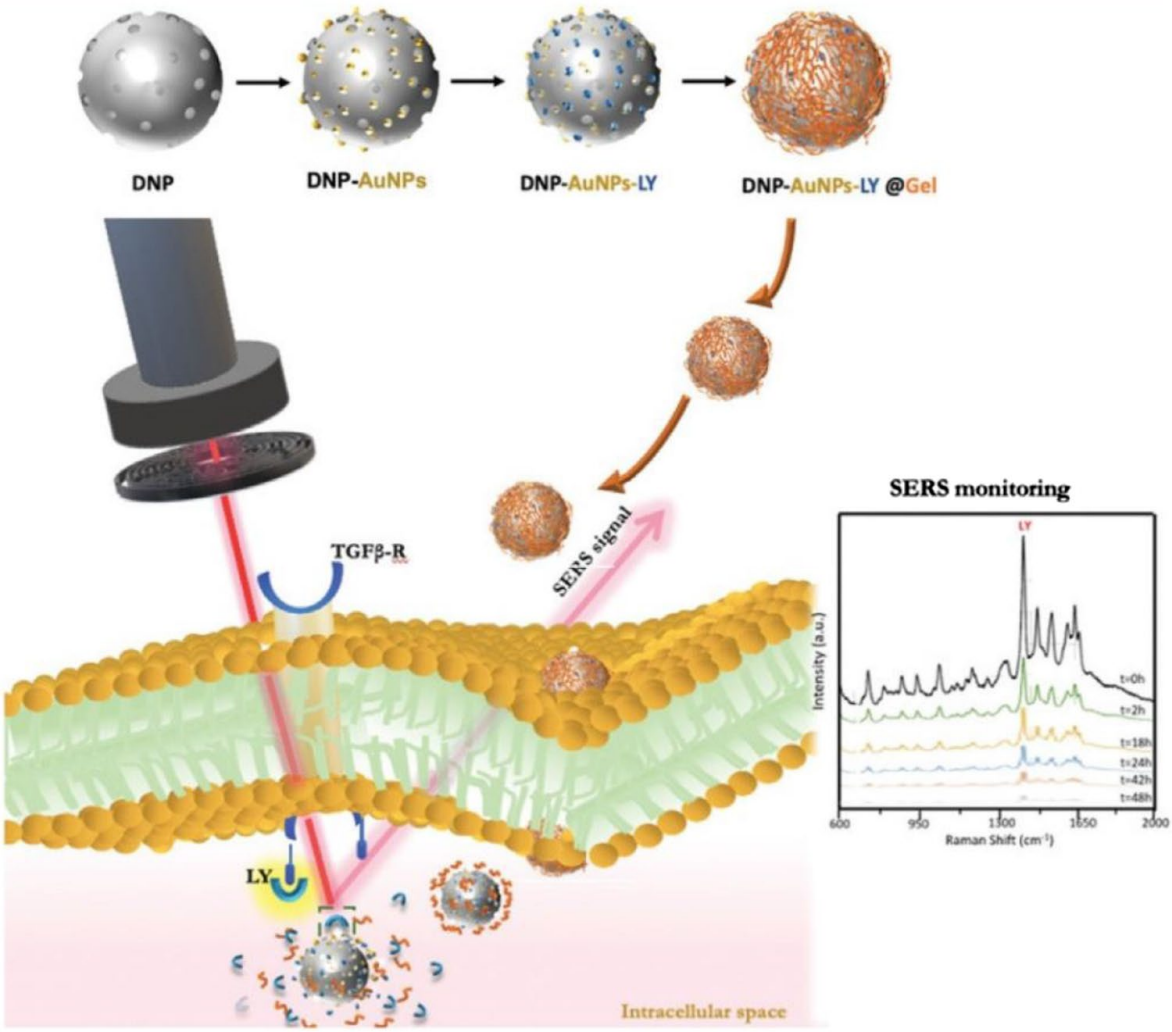
Schematic illustration of the synthesis and internalization of gold-coated diatomite nanoparticles encapsulated with a gelatin layer within a CRC cell. The acidic tumor microenvironment triggers the degradation of the gelatin shell, leading to the release of the encapsulated drug. SERS was used to quantify the amount of drug released as described by Manago et al. (Managò et al. 2021). Reproduced with permission from Managò et al. (2021); Zobi 2022

Tramontano et al. further improved these carriers by incorporating gold-coated, gelatin-capped diatomite nanoparticles (Tramontano et al. 2021). This design allows for a controlled and increased drug-loading capacity by adjusting the thickness of the gelatin shell surrounding the biosilica core. Gold coating adds another layer of functionality by enabling label-free, real-time drug release monitoring via near-field optical amplification of surface plasmon resonance (NFOAS). This highly sensitive and specific technique utilizes shifts in the resonance of gold nanoparticles to track changes in the gelatin shell, reflecting both its degradation and subsequent drug release. This research focused on delivering galunisertib, an anticancer drug targeting the transforming growth factor-β1 (TGF-β1) receptor (Managò et al. 2021; Sivashanmugan et al. 2019) (as shown in Fig. 9). This receptor plays a crucial role in epithelial-to-mesenchymal transition (EMT), which enables cancer cells to acquire a more invasive and metastatic phenotype, particularly in CRC (Holmgaard et al. 2018). The pH-sensitive release of galunisertib from diatomite nanoparticles, facilitated by the acidic microenvironment of CRC cells, promotes antimetastatic effects by inhibiting EMT. This study highlights the potential of these multifunctional nanoparticles for targeted drug delivery with real-time monitoring capabilities.

Javalkote et al. used the ferrofluid method or in situ NP synthesis to incorporate iron oxide nanoparticles (FeONPs) into *Nitzschia* spp. to create magnetically active and responsive diatom frustules (Javalkote et al. 2015). Curcumin, a model chemotherapeutic agent, was injected into these magnetic diatoms, demonstrating its potential as a drug delivery vehicle. The authors did not investigate how an external magnetic field affected the ability of the diatoms to release drugs, even though they were magnetically responsive. Nevertheless, this idea is worth exploring further in the future (Kabir et al. 2020). Uthappa et al. loaded curcumin onto polydopamine (PDA) surface-modified diatoms and examined the impact of PDA on its drug-loading and drug-releasing characteristics. Compared to unmodified diatoms, they discovered that PDA functioned as a barrier, lowering the drug discharge rate by approximately 15–20%. This implies that PDA surface modification might be a workable method for obtaining medication delivery systems ready for targeted cancer treatment (Uthappa et al. 2019). Finally, Vona et al. investigated the incorporation and delivery of the fungal phytotoxic-derived chemical Ophiobolin A (OphA), a broad-spectrum chemotherapeutic agent that is efficient against a variety of cancer cell lines, using unmodified diatomaceous earth. Notwithstanding the limitations of this work, it was suggested that diatomite activated by acid-oxidative treatment could function as a loading/delivery mechanism for OphA, prolonging the duration of drug release and increasing the total absolute amount of drug released, which would be equivalent to the half-life of OphA (Cicco et al. 2015).

### Targeted inorganic therapeutics

**Fig. 10:**
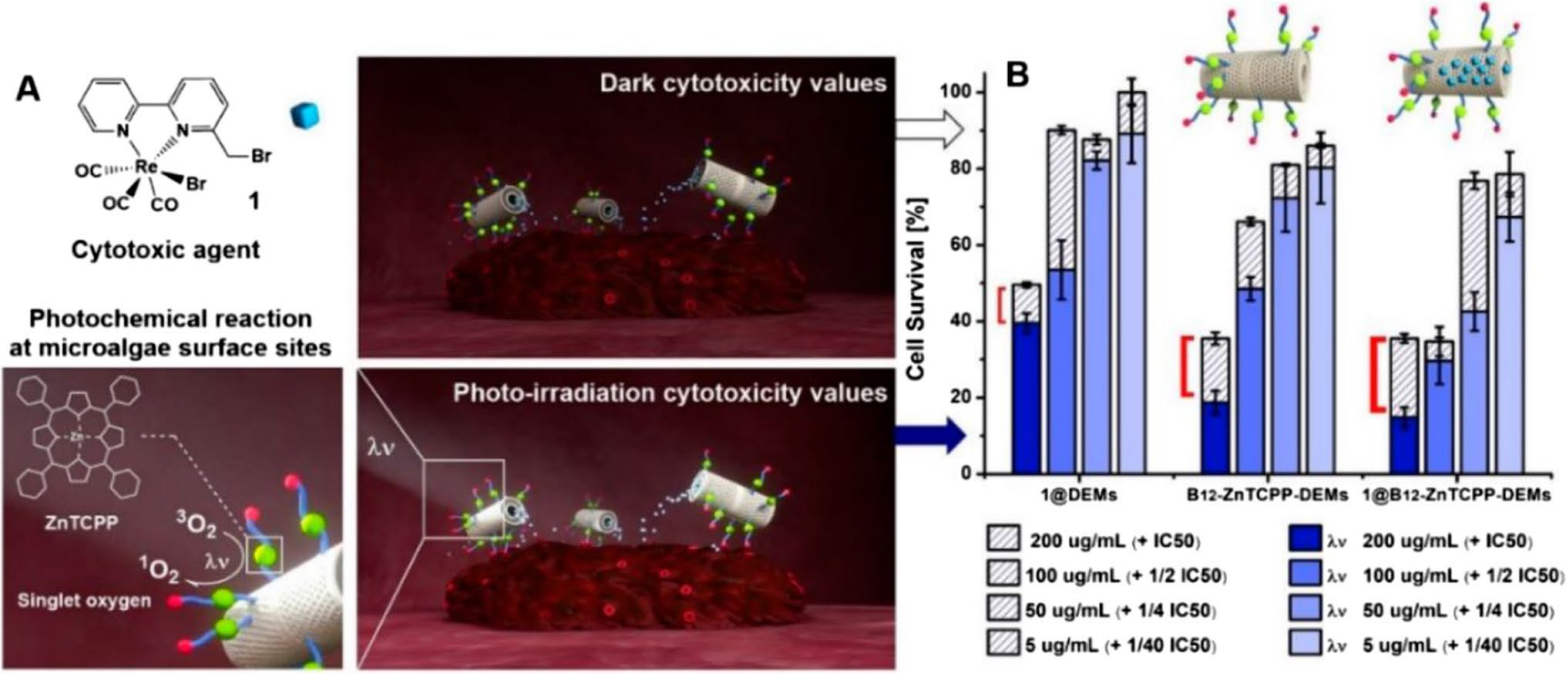
Conceptual depiction of photoactivatable surface-functionalized diatoms designed by Delasoie et al. (Delasoie et al. 2020) to enhance the cytotoxicity of anticancer complexes. **A** Structure of the anticancer complex and details the photochemical reactions that occur on the diatom surface. **B** Histograms demonstrating the effect of the material on HCT-116 CRC cell viability at varying doses. Cells treated in the dark (empty bars) were compared to those exposed to light activation of the diatom surface (filled bars). The bracketed region on the graph highlights the significant increase in cytotoxicity upon light irradiation (refer to Delasoie et al. (2020) for permission to reproduce the figure)

Researchers Delasoie et al. demonstrated the surface functionalization of diatomaceous earth microparticles with vitamin B12, which serves as a tumor-targeting drug, as well as the assessment of the loading and release performance of a polypyridyl ruthenium (II) complex, 5-fluorouracil (5-FU), and cisplatin. The lipophilic ruthenium complex showed a distinct release profile, whereas cisplatin and 5-FU were quickly released from the material. In aqueous media, the chemical was maintained in the diatoms for up to five days but in lipophilic environments, such as the cell membrane (Delasoie et al. 2018; Rossier et al. 2017). The greater adherence of vitamin B12-coated particles to HT-29 CRC cells was significant and correlated with greater expression of TC(II) and TC(II)-R in the tissues. This discovery may impact the potential use of this substance for targeted medication administration. Researchers have further enhanced their idea by using diatom microalgae with photoactivatable surface functionalization to increase the cytotoxicity of certain anticancer compounds. They specifically selected very potent rhenium compounds known for their ability to combat bacteria and CRC. The results showed that photoactivating the surface of the microalgae significantly increased the overall toxicity of the hybrid multifunctional drug delivery system, potentially doubling the cytotoxic efficacy of the loaded medication. Additionally, Delasoie et al. examined the neovascularization effects of medicines that release carbon monoxide and are chemically attached to *Coscinodiscus* diatom carriers (Delasoie et al. 2018). The administration of these rhenium complexes at concentrations ≥ 25 µM significantly reduced the formation of intersegmental and sub intestinal capillaries in an *in vivo* zebrafish model (Sovari et al. 2021, 2020), suggesting a substantial antiangiogenic effect. Finally, Shi et al. reported silica-protein nanocomposites that resembled diatoms and were created for the sustained delivery of polypyridyl ruthenium (II) complexes (Shi et al. 2021) (Fig. 10). These nanocomposites were made from tiny silica nanoparticles around human serum albumin, emulating the structure of diatoms. By enabling clathrin-mediated endocytosis of lysosomes, they allowed the inorganic drug to concentrate intracellularly and release itself under regulated conditions. Reactive oxygen species could be produced by light irradiation of the composite material, exhibiting remarkable efficacy in photodynamic treatment. The authors found that diatom-like nanostructures might be useful for photodynamic treatment, bioimaging, and sustained drug delivery nanocarrier applications.

### Biodelivery of antibiotics

**Fig. 11:**
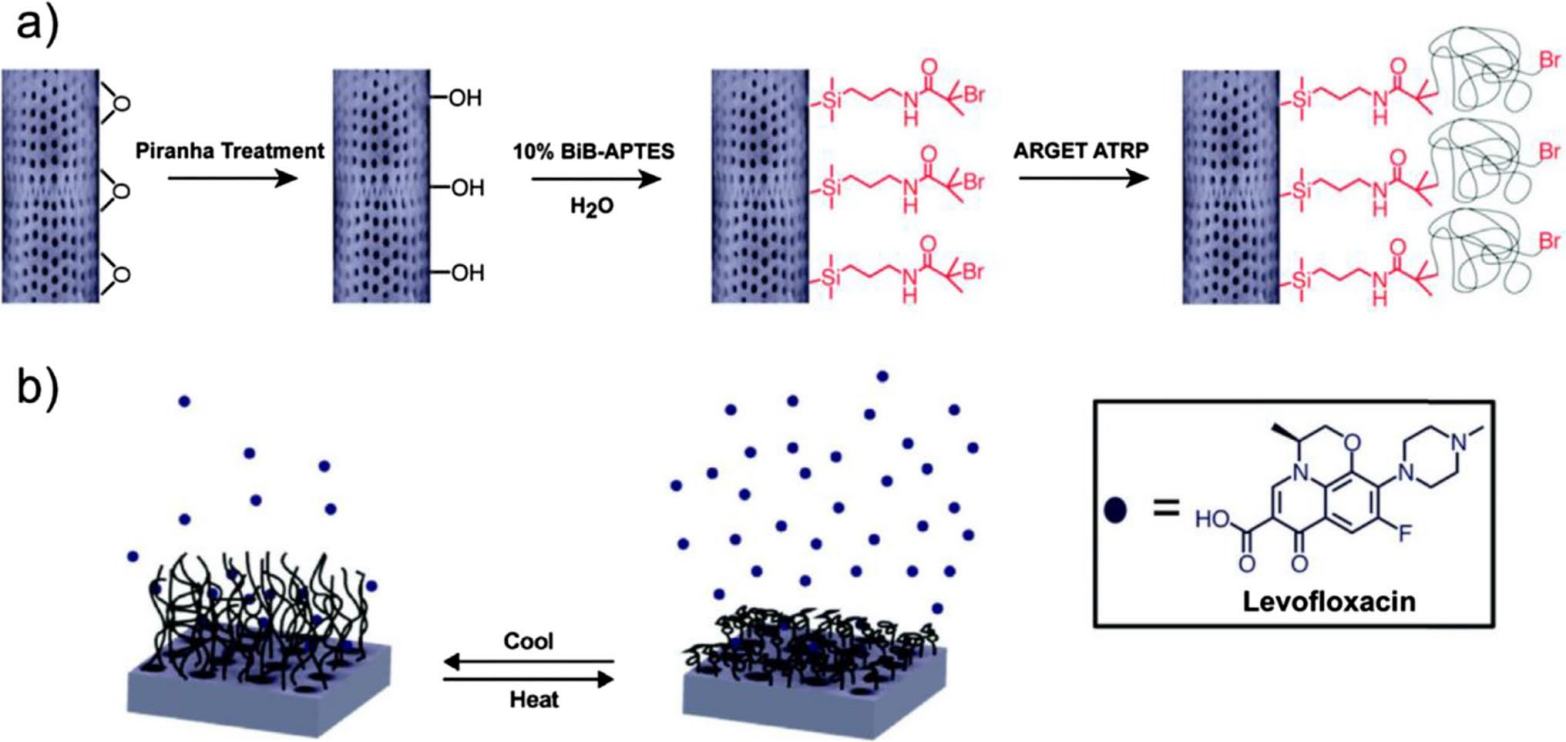
Schematic illustration of **A** the functionalization process of diatom biosilica microcapsules and **B** subsequent drug release from the thermoresponsive polymer-grafted biosilica frustule. Reproduced with permission from Vasani et al. (2015)

Few studies have examined the potential of diatoms as antibiotic delivery systems in comparison with those concentrating on their application in cancer therapy experiments. Vasani et al. (2015) presented a method for creating stimulus-responsive diatom biosilica microcapsules to deliver levofloxacin (Vasani et al. 2015). By employing silanization chemistry, they were able to alter the thermoresponsive *Aulacoseira* spp. diatoms and adhere oligo (ethylene glycol) methacrylate copolymers to their surface. This modification enabled the release of the antibiotic under temperature control, which could be useful for treating inflamed infection sites. Scientists have shown that these altered diatoms loaded with levofloxacin have thermoresponsive antibacterial activity against *Pseudomonas aeruginosa* and *Staphylococcus aureus* (as shown in Fig. 11).

**Fig. 12:**
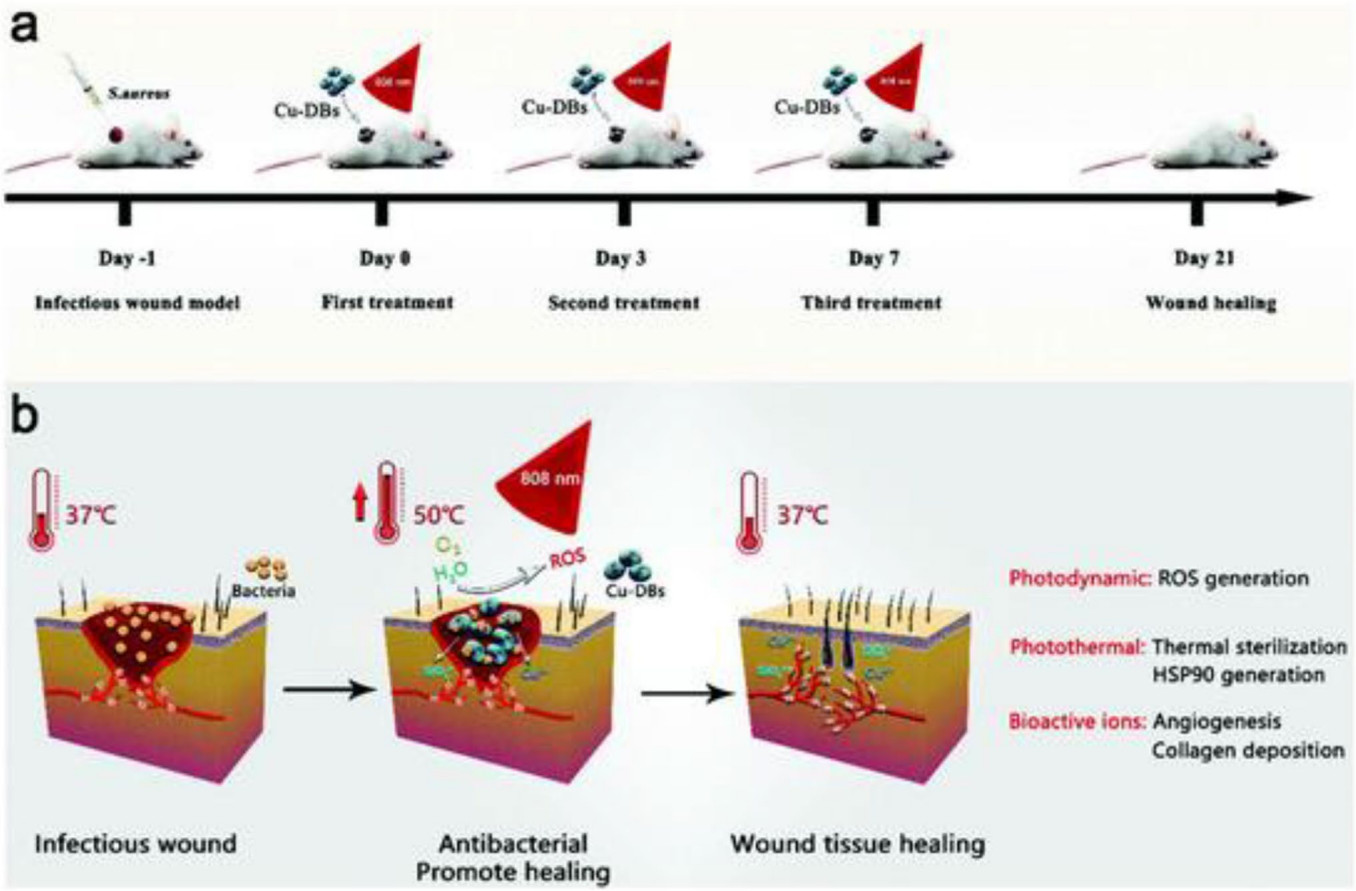
Copper-deposited diatom biosilica was used for enhanced infected wound healing. **A** The treatment protocol. **B** The proposed mechanism of wound healing. Reproduced with permission from Cong et al. (2022)

Cicco et al. (2015) investigated the ability of *Thalassiosira weissflogii* diatoms to carry the antibiotic ciprofloxacin and provide anti-inflammatory protection against its side effects. They attached 2,6,6-tetramethylpiperidine-N-oxyl (TEMPO), a cyclic nitroxide that functions as a scavenger of reactive oxygen species (ROS), to the diatom surface. The modification process used carbodiimide-activated esterification to bind 4-carboxy-TEMPO with 3-aminopropyl triethoxy silane. Although diatom-based systems typically release pharmaceuticals in bursts within the first 24 h, only 17–24% of the loaded ciprofloxacin was released after 7 days in saline or PBS media, indicating sustained release characteristics. Additionally, the modified biosilica promoted cell growth and showed biocompatibility with MG63 osteoblast-like cells, suggesting potential applications for bone cells in regenerative medicine (Cicco et al. 2015). In a more recent study, Uthappa and colleagues reported diatom biosilica-MOF hybrid materials for delivering the antituberculosis (TB) drug isoniazid (INH) (Uthappa et al. 2020). These materials, known as “MIL-100(Fe)-DE,” exhibited enhanced drug loading capacity and prolonged, controlled release of the drug over 23 days. This extended release is beneficial because INH has a short biological half-life (1–4 h), which limits its therapeutic benefits in TB treatment. Briceno and colleagues decorated diatoms of the *Aulacoseria* genus with gold nanoparticles (AuNPs) and studied the *in vitro* release of gentamicin in simulated body fluid. They discovered that different techniques for AuNP deposition led to variable release rates and efficiencies, highlighting the versatility of diatoms in controlled drug delivery applications (Briceño et al. 2021). An example, Copper-deposited diatom biosilica was used for enhanced infected wound healing (as shown in Fig. 12).

### Alternating drug delivery strategies

Only two studies that used diatomite for drug release, including drugs not previously covered, were conducted throughout the reviewed period. Diatomaceous earth microparticles modified with xerogel (DE-XER) were created by Uthappa et al. to regulate diclofenac sodium, an anti-inflammatory and analgesic medication. Because DE-XER operates as a pH-sensitive microcarrier, drug release can be regulated and controlled. Compared with unmodified DE, the covalent xerogel coating increased the surface area and pore volume of the material, enhancing medication absorption (Uthappa et al. 2018). This suggests that diffusive transport through precisely calibrated porous structures, influenced by the material’s surface characteristics, is the primary mechanism underlying drug release (Uthappa et al. 2018; Sherief et al. 2021). Resveratrol is an anti-inflammatory and antioxidant medication released by Bonifacio et al. that uses gellan gum, honey, and diatom composite materials. These porous composite scaffolds were created with cartilage regeneration in mind, providing appropriate mechanical and substantial antimicrobial qualities, articular load-bearing qualities, and regulated resveratrol release. This regulated release promoted chondrogenic differentiation and stem cell colonization while efficiently suppressing bacterial growth (Bonifacio et al. 2020). Recently, diatom formulations have been investigated for tissue engineering applications, and some of these applications have also been examined for drug delivery systems. Diatom silica/polysaccharide elastomeric hydrogels were characterized by Lee et al. as water-resistant, sticky tissues with shock-absorbing capabilities. In an ischemia‒reperfusion-based pressure ulcer animal model, these hydrogels, which were created by the oxidative crosslinking of catechol and chitosan on diatom surfaces, aided in the early stages of the healing process (Lee et al. 2021). In a study using a rat tail amputation model, Feng et al. demonstrated exceptional erythrocyte absorption and aggregation, as well as coagulation activation, by utilizing chitosan-coated *Coscinodiscus* spp. diatoms as a hemostatic agent for managing hemorrhage (Feng et al. 2016). Other researchers have utilized diatom-inspired techniques for various purposes, such as mineralizing the surface of insulin, designing tools to evaluate the influence of surface topography on cell fate, developing pollen-like surface patterns, and examining the mechanical response of diatom-inspired structures (Gutiérrez et al. 2018; Walsh et al. 2017; Krishna et al. 2021).

## Marine diatoms: applications and advancements

Marine diatoms, a diverse group of microalgae, are known for their ability to synthesize and accumulate lipids. With the increasing global demand for sustainable energy sources and the depletion of fossil fuel reserves, there has been growing interest in exploring alternatives, and marine diatoms represent renewable and abundant resources in this regard. These lipids are mainly triglycerides (TAGs), which serve as energy storage molecules within diatom cells. The lipid content can vary significantly depending on factors such as species and growth rate. Polyunsaturated fatty acids (PUFAs) are essential nutrients with numerous health benefits, including cardiovascular support. The marine environment provides diatoms with an abundance of nutrients and light, providing suitable conditions for lipid biosynthesis. Some species, such as *Thalassiosira phaeodactylum*, are used for lipid extraction (Stanković et al. 2022). The extraction of lipids from marine diatoms is both a challenge and an opportunity. These methods are sensitive to oxidation and degradation and require careful extraction processing techniques. Several methods, such as extraction methods, solvent-based approaches, and enzymatic hydrolysis, have been developed to efficiently extract PUFAs from diatom biomass while minimizing lipid oxidation and degradation.

### Lipid extraction procedure

**Fig. 13:**
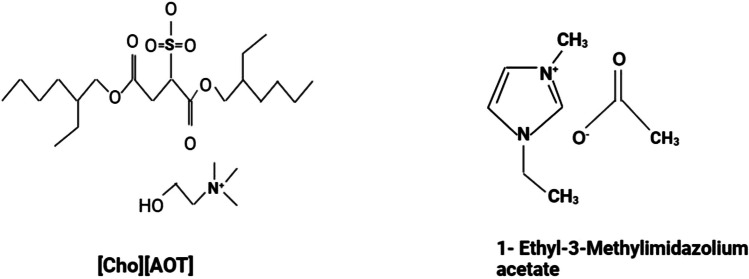
The structure of choline dioctyl sulfosuccinate ([Cho][AOT]) and 1-ethyl-3-methylimidazolium acetate

Choline dioctyl sulfosuccinate ([Cho][AOT]) was synthesized by dissolving equimolar amounts of sodium dioctyl sulfosuccinate and choline chloride in water, followed by agitation at room temperature for one day (the structures are shown in Fig. 13). The advancement of the reaction was observed by thin layer chromatography.

**Fig. 14:**
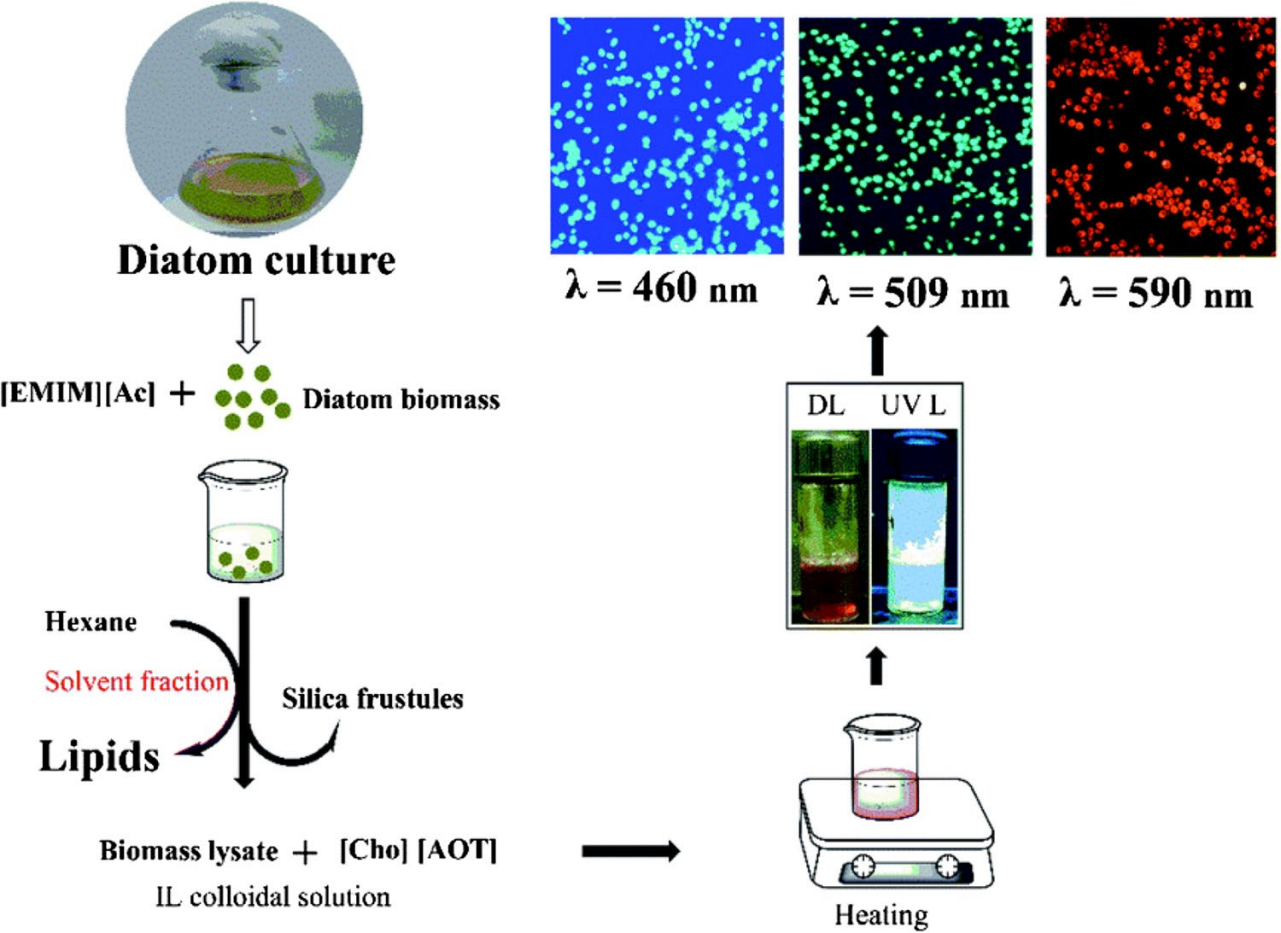
Schematic representation of lipid extraction and hydrothermal methods for synthesizing green, fluorescent carbon dots from biomass residue and their application. Reproduced with permission from Stanković et al. (2022)

Once the reaction was finished, the product was isolated from the dimethane chlorine layer and washed with water to eliminate any remaining 1 M AgNO_3_, leading to a transparent solution. The dichloromethane layer devoid of chloride was then distilled to obtain pure [Cho][AOT], which underwent vacuum drying and was kept in a desiccator. The purity of [Cho][AOT] was validated through liquid chromatography–mass spectrometry (LC‒MS) and 1H nuclear magnetic resonance (NMR) analyses. Furthermore, diatom *Thalassiosira lundiana* cultures were kept for fifteen days in modified f/2 media in a five-liter culture flask with 2.5 L of media. Specific nutrients were added to the medium, and the number of cells was checked every three days at 25 ± 1 °C. The diatom was removed from the culture, freeze-dried, mixed with an ionic liquid (imidazole), and ultrasonicated to rupture the cells to identify the lipids in the culture. The mixture was collected following the extraction of lipids using hexane. N,O-Bis(trimethylsilyl)trifluoroacetamide (BSTFA) is utilized to vaporize the collected lipids and form derivatives. Gas chromatography‒mass spectrometry is utilized for the identification of the fatty acid varieties present. Software was used for analyzing the obtained fatty acids, and a reference library was used for comparison. The method involves using an ionic liquid-based solvent solution to extract lipids from a specific type of diatom. After growing exponentially, these diatoms eventually enter a stationary phase due to nutritional depletion. Fatty acids are released from microalgae by breaking down their cell wall using an ionic liquid-based solvent solution. Using various assays that showed the ionic liquid was appropriate for lipid extraction, its stability was limited (schematically represented in Fig. 14).

## Diatom biosensors: pioneering ecological monitoring

The use of diatoms in ecological biosensing has gained attention in recent years due to their unique parcels and implicit operations in environmental monitoring. Diatoms are microorganisms that are largely sensitive to changes in their girding terrain, making them ideal pointers for water quality and ecosystem health. By employing the capabilities of diatoms, experimenters can develop innovative biosensing technologies to track and cover pollution situations in gutters, lakes, and abysses (Rosandi et al. 2024).

### Biosensing technologies exercising diatoms

Advancements in biosensing technologies have decreasingly employed diatoms due to their unique parcels. Diatoms, known for their intricate silica shells, offer an ideal platform for biosensor development. By employing natural photonics and face parcels of diatoms, experimenters have been able to produce largely sensitive and specific biosensors for a range of operations. These biosensors have been employed in environmental monitoring, medical diagnostics, and food safety, among others (Nadumane et al. 2024). The integration of diatoms into biosensors has opened new avenues for enhancing discovery limits and selectivity, making them valuable tools in colorful fields. Overall, biosensing technologies for exercising diatoms highlight the success of nature-inspired design in developing advanced-seeing platforms.

### Diatom operations in environmental monitoring

The use of diatoms in environmental monitoring has gained significant traction in recent years due to their perceptivity to environmental changes. By exercising diatoms as bioindicators, experimenters can assess water quality, pollution situations, and ecosystem health. The response of diatoms to colorful stressors, such as temperature oscillations or nutrient vacuity, makes them valuable tools for covering environmental conditions. Additionally, diatom-grounded biosensors offer a cost-effective and dependable system for the nonstop monitoring of water bodies by detecting shifts in ecological parameters instantly (Temraleeva and Sinetova 2023). These biosensors are designed to measure changes in diatom populations directly, furnishing real-time data pivotal for effective environmental operation and conservation of sweats. The perpetuation of diatoms in environmental monitoring not only enhances our understanding of ecosystem dynamics but also aids in the early discovery of environmental disturbances, contributing to sustainable environmental practices and policymaking. Further exploration and technological advancements in diatom-grounded monitoring systems can revolutionize environmental studies, paving the way for further effective ecological biosensing strategies (Visco et al. 2015). Diatoms are crucial in oxygenic photosynthesis by sequestering carbon in their structures, which sink to the seafloor, removing carbon from the cycle. This process increases oxygen levels and decreases CO_2_ concentrations, supporting the evolution of larger marine organisms (Falciatore and Bowler 2002). Diatom’s calcification and silicification contribute to carbon sequestration, forming sedimentary rocks and supporting the evolution of larger marine organisms. Their high productivity and abundance support diverse marine food chains, contributing significantly to biodiversity (Sethi et al. 2020). Diatoms’ membranes contain specialized structures for light capture, including stacks and fucoxanthin pigment. They also use a protein called fucoxanthin-chlorophyll proteins (FCP) for optimal light capture. Some diatoms use C_4_ photosynthesis mechanisms to efficiently store carbon for future use. As a byproduct, diatoms release oxygen, essential for marine organism survival. Diatoms significantly impact the global carbon cycle by assimilating CO_2_ from the atmosphere and ocean waters, converting it into organic carbon compounds (Purcarea et al. 2024).

### Case studies of diatom-grounded biosensors

Case studies of diatom-grounded biosensors have demonstrated their efficacy in colorful ecological operations. For this purpose, a study conducted by Cristina Purcarea et al. used diatom-grounded biosensors to assess water quality in brackish ecosystems. These biosensors, which represent the unique responses of diatoms to environmental stimulants, are suitable for directly describing changes in nutrient conditions and pollution, providing real-time data for effective operation strategies (Purcarea et al. 2024). Similarly, another case study by Johnson et al. showed the ability of diatom-ground biosensors to detect heavy impurities in marine surroundings. Using specific diatom species known to accumulate heavy essence, experimenters identified hotspots of pollution and prioritized the remediation of sweats. These examples highlight the versatility and perfection of diatom-grounded biosensors, making them inestimable tools for ecological monitoring and conservation of sweats (Dhanker et al. 2023).

## Conclusions

Diatoms are vital microorganisms in aquatic ecosystems, contributing to primary production of organic matter and also the carbon cycle. Their unique silica cell walls offer structural stability and protection from desiccation and predation. Classification based on morphological characteristics helps understand their diversity and distribution. Advances in molecular techniques reveal genetic relationships among different diatom species. Diatoms offer a promising platform for the development of biosensors due to their high surface area, biocompatibility, and ease of functionalization (De and Mazumder 2022). The economic importance of diatoms extends beyond their biosensing applications, encompassing a wide range of industries such as abrasives, food, and pharmaceuticals. The potential of diatoms in optical biosensing is significant, with ongoing studies focusing on enhancing their sensitivity, selectivity, and stability (Blanco 2024). Genetic engineering and surface modifications are key areas of exploration, aiming to tailor diatom biosilica for specific sensing applications. Research into diatoms and biosensing holds promise for healthcare, environmental monitoring, and materials science. Diatoms are valuable indicators of ecosystem health due to their rapid response to environmental changes and high perceptivity to adulterants (Taurozzi et al. 2024). Integrating diatom-grounded biosensors with advanced technologies like molecular methods and remote sensing can further enhance environmental assessment. Diatoms have been exploited since the 1980s as a source of natural biosilica for the blueprint development of new generation of drug delivery systems and biosensing materials (Fu et al. 2022). Their unique 3D structure, biocompatibility, and ability to be integrated into hybrid systems make them ideal for various biosensing applications, including drug delivery, environmental monitoring, and medical diagnostics. Although much is to be discovered regarding diatom cell morphology and adaptation, recent work has demonstrated their values in biotechnology applications (Saxena et al. 2021). In drug delivery, diatom frustules can be functionalized with different chemical groups to create targeted delivery systems. Marine diatoms hold ecological significance, contributing to the ocean's primary productivity and nutrient cycling. Efficient lipid extraction techniques from diatoms can enhance the viability of large-scale biofuel production (Tommasi and Luca 2022). In conclusion, the applications of diatoms range from medical diagnostics to environmental monitoring, highlighting their broad potential and importance in advancing biosensing technologies.

## Data Availability

Data will be available upon request to the corresponding author.
